# Synaptotagmin-7, a binding protein of P53, inhibits the senescence and promotes the tumorigenicity of lung cancer cells

**DOI:** 10.1042/BSR20181298

**Published:** 2019-02-08

**Authors:** Zhenhua Fei, Wei Gao, Raoying Xie, Ganzhu Feng, Xiaolin Chen, Yiyan Jiang

**Affiliations:** 1 Department of Radiation and Medical Oncology, The 1st Affiliated Hospital of Wenzhou Medical University, Wenzhou 325000, P.R. China; 2 Department of Geriatric Medicine, Sir Run Run Hospital, Nanjing Medical University, Nanjing 211100, P.R. China; 3 Department of Respiratory Medicine, Sir Run Run Hospital, Nanjing Medical University, Nanjing 211100, P.R. China; 4 Department of Tumor Rehabilitation, The 1st Affiliated Hospital of Wenzhou Medical University, Wenzhou 325000, P.R. China

**Keywords:** Lung cancer, P53, Senescence, SYT7

## Abstract

Lung cancer has been one of the most common malignancies in the world. Cell senescence has been recognized as the avenue to inhibit tumor progression. However, the mechanisms remain poorly understood. In the present study, we have shown that synaptotagmin-7 (SYT7) expression was up-regulated in lung cancer. SYT7 also promoted the growth and colony formation of lung cancer cells and inhibited their senescence. In a molecular mechanism study, SYT7 was shown to interact with P53 and to potentiate the interaction between P53 and MDM2. Taken together, the present study demonstrates the oncogenic roles of SYT7 in lung cancer, and suggests that SYT7 may be a good therapeutic target for lung cancer treatment.

## Introduction

Combining both sexes worldwide, lung cancer is the most commonly diagnosed cancer and the leading cause of cancer death [1]. The incidence of lung cancer is increasing. Surgical resection has been recognized as the best method for the treatment of lung cancer [2]. Although chemotherapy and radiotherapy are widely used, the therapeutic resistance of lung cancer cells is the main reason for treatment failure. Therefore, a better understanding of the molecular mechanisms of this malignancy will help the development of a successful therapy.

The development of many open data resources has provided an opportunity for researchers to analyze the significance of differentially expressed genes in lung cancer. By analyzing the GEPIA database [3], we found that synaptotagmin-7 (SYT7) was highly expressed in lung cancer (http://gepia.cancer-pku.cn/detail.php?gene=SYT7). Therefore, we chose SYT7 as a candidate gene for further research.

STY7 mediates the calcium-dependent regulation of membrane trafficking during synaptic transmission [4–6]. Several studies have demonstrated the oncogenic role of SYT7 in tumorigenesis [7–9]. SYT7 promoted the proliferation of colon cancer cells and glioblastoma cells [7]. In gastric cancer, SYT7 has been demonstrated to act as a driver for metastasis formation [8]. However, the function of SYT7 in lung cancer remains unknown.

Cellular senescence induces cell growth arrest when cells are subjected to cellular stress [10]. Numerous studies have indicated that cell senescence was an important tumor-suppressor mechanism [11]. P53, P21, P16, and retinoblastoma protein (Rb) have been recognized as the major regulators of cell senescence [12]. Therefore, mutations of P53 or down-regulation of P53 expression, by up-regulating its ubiquitin ligase MDM2, have been shown to overcome cell senescence and lead to therapy resistance [13].

In the present study, we have examined the expression of SYT7, investigated its functions and explored its molecular mechanisms.

## Materials and methods

### Cell culture

Lung cancer cell lines (H23, H520, SPAC-A-1, and A549) and normal lung epithelial cells (BEASE-2B) were obtained from the Cell Bank of Shanghai Institutes for Biological Science. Cells were maintained in DMEM medium supplemented with 10% fetal bovine serum (GIBCO), 100 units/ml of penicillin and 100 g/ml of streptomycin, in an incubator with 5% CO_2_ at 37°C.

### Clinical samples

Lung cancer samples and paired noncancerous tissues were collected from patients who underwent surgery at Sir Run Run Hospital, Nanjing Medical University, after obtaining the consent of the patients. Collected tissues were stored in liquid nitrogen. The present study was approved by the Ethics Committee of our institution.

### Western blot analysis

The proteins were extracted from tissues and cell lines using the RIPA lysis buffer and were separated by SDS-PAGE. Then, the proteins were transferred onto a polyvinylidene fluoride (PVDF) membrane. After blocking with 5% of BSA solution for 1 h at room temperature, the membrane was incubated with the primary antibodies overnight. Then, the membrane was washed with TBST solution and incubated with the secondary antibody for 1 h at room temperature. The proteins were visualized using an ECL kit.

### Immunohistochemistry

The sections were deparaffinized and rehydrated using xylene and ethanol, then a 0.3% H_2_O_2_ solution was used to block the endogenous peroxidase activity. Afterward, the antigens were retrieved using sodium citrate solution (pH 6.0) and nonspecific binding of SYT7 antibody was blocked using 5% of BSA solution. Next, the sections were stained with SYT7 antibody and visualized with the secondary antibody (Envision, Gene Technology). Then, the slides were developed with DAB and counterstained with hematoxylin.

### GST pull-down

The coding sequence of P53 was cloned into the expression vector pGEX-4T-1, and the fusion protein, GST-P53, was purified. H23 whole cell lysates were prepared using 50 mM of Tris-Cl (pH 7.5), 150 mM of NaCl, 0.1% of NP40, and a protease inhibitor cocktail. Then, 5 μg of the GST-P53 fusion protein and 500 μg of cell lysates were incubated overnight at 4°C. Afterward, 50 μl of Glutathione Sepharose 4B beads was added to the samples and incubated at 4°C for 1 h to capture the GST fusion proteins. After washing three times with lysis buffer, the proteins were eluted in Laemmli buffer and analyzed by SDS-PAGE.

### Immunoprecipitation assay

For the immunoprecipitation assay, cells were lysed with RIPA buffer. After centrifugation at 4°C for 20 min (12000 ***g***), the supernatant of the cell lysate was incubated with the primary antibody overnight at 4°C. Then, the supernatant was incubated with the protein A beads for another 4 h, and the beads were washed three times with RIPA buffer. Finally, loading buffer was added to the immunoprecipitate solutions that were boiled for 5 min at 100°C, and subjected to Western blot.

### Plasmids

The coding sequence of SYT7 was amplified by PCR and inserted into the expression vector pcDNA3.1 to obtain the myc-tagged SYT7. The coding sequence of MDM2 was amplified by PCR and inserted into the expression vector pCMVTag2B to obtain the Flag-tagged MDM2. The coding sequence of P53 was amplified by PCR and inserted into the expression vector pCMV-HA to obtain the HA-tagged P53.

### Knockdown of SYT7 expression

The RNAi lentivirus particles (sh con and sh SYT7) were purchased from GeneChem (China). Cells were infected with the indicated lentivirus particles of the same MOI for 24 h, and stable knockdown cells were selected with the medium containing puromycin for at least 1 week.

### MTT assay

Cells were plated in 96-well plates at a density of 10^3^ cells/well. Cell growth was determined by 3-(4,5-methylthiazol-2-yl)-2,5-diphenyltetrazolium bromide (MTT) colorimetric growth assay for 1 week. Every other day, cell growth was determined by incubation with the MTT solution (50 μg/well) for 4 h. Cellular MTT was resolved with DMSO and measured at 540 nm. All experiments were performed in triplicate.

### Soft agar assay

For the soft agar assay, 2 × 10^3^ cells/well were suspended in the soft agar upper layer (0.35% agarose and 10% FBS in DMEM) and plated in 12-well plates; the plates were then coated with the soft agar bottom layer (0.5% agarose and 10% FBS in DMEM). After 14 days of incubation, the colonies were counted and measured. All experiments were performed at least three times.

### Statistical analysis

Statistical analysis was performed by Student’s *t*-test (two-tailed) using the Prism GraphPad software. *P*<0.05 were considered statistically significant. Data were presented as the mean ± standard error of the mean (SEM).

## Results

### The expression of SYT1 was elevated in lung cancer

**Figure 1:**
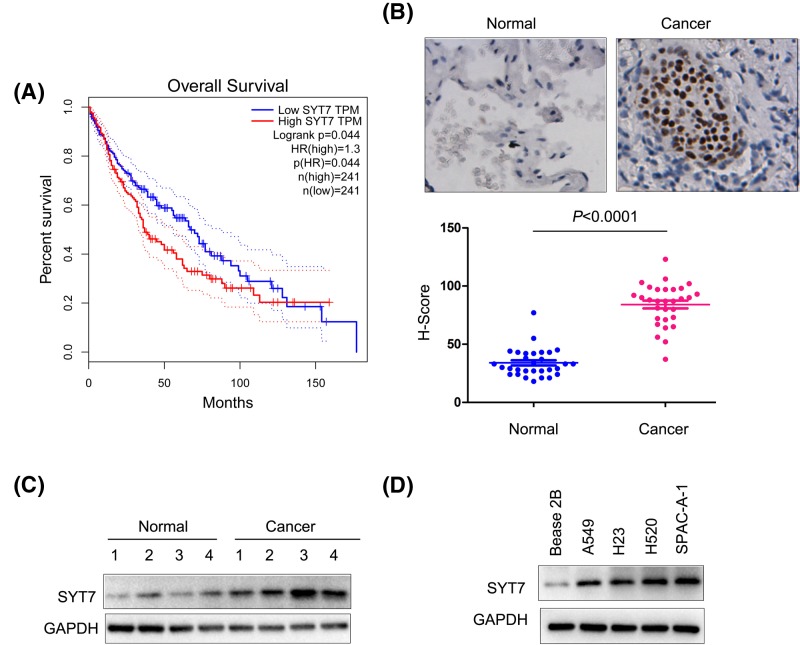
SYT7 was up-regulated in lung cancer (**A**) Mining the GEPIA database to examine the correlation between SYT7 expression levels and survival. The cohort included 482 patients and was divided into two groups according to the expression of SYT7 (high vs low). (**B**) The IHC was performed to examine the expression of SYT7 in lung cancer tissues and adjacent normal tissues. (**C**) Western blot analysis was performed to examine the expression of SYT7 in lung cancer tissues and noncancerous tissues. (**D**) Western blot analysis was performed to examine the expression of SYT7 in lung cancer cells (A549, H23, SPAC-A-1 and H520) and normal lung cells (Bease-2B).

We first turned to the GEPIA database to explore the expression pattern of SYT7 in lung cancer. As shown in Figure 1A, higher SYT7 expression was associated with poor survival (Figure 1A). We next assessed the expression of SYT7 in lung cancer tissues and paired adjacent normal tissues by using immunohistochemistry (IxHC) and Western blot analysis. We found that SYT7 protein levels in lung cancer tissues were elevated (Figure 1B,C). In addition, using a series of lung cancer cell lines (A549, H23, H520, SPAC-A-1) and normal lung cells (Bease-2B), we found higher expression level of SYT7 protein in lung cancer cell lines (Figure 1D). These observations suggested that SYT7 was up-regulated in lung cancer.

### SYT7 promoted the growth and colony formation of lung cancer cells while inhibiting their senescence

**Figure 2:**
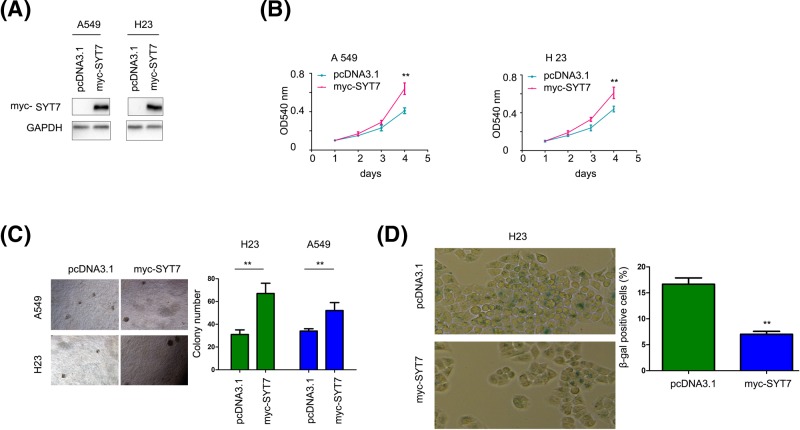
SYT7 promoted the growth of lung cancer cells (**A**) Western blot was performed to confirm the expression of myc-SYT7 in A549 and H23 cells. (**B**) MTT assay was performed to examine the growth of lung cancer cells. (**C**) Colony formation assay was performed to examine the growth of lung cancer cells on soft agar. (**D**) Beta-gal staining was performed to examine the senescence of lung cancer cells; **, *P*<0.01.

To examine the functions of SYT7 in lung cancer, we forced the expression of SYT7 in A549 and H23 cells (Figure 2A). Overexpression of SYT7 in lung cancer cells promoted the growth of A549 and H23 cells, not only in liquid cultures but also in soft agar (Figure 2B,C). Moreover, more beta-gal positively stained cells were found in control cells compared with SYT7 overexpressing cells (Figure 2D), suggesting that SYT7 inhibited cellular senescence.

**Figure 3:**
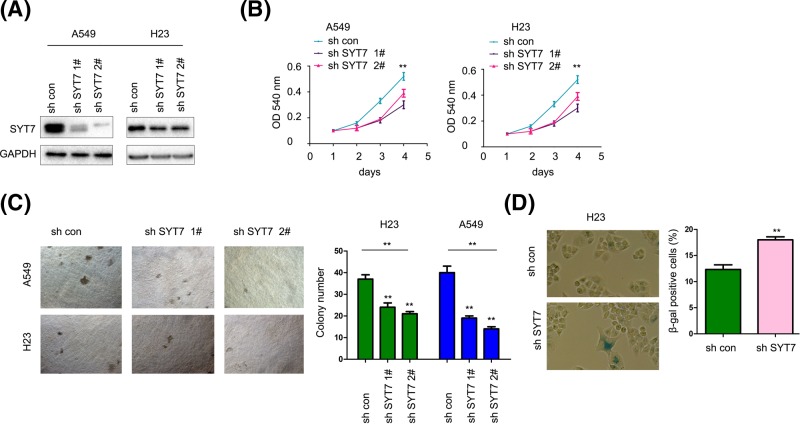
Knocking down of SYT7 inhibited the growth of lung cancer cells (**A**) Western blot was performed to confirm the down-regulation of SYT7 in A549 and H23 cells. (**B**) MTT assay was performed to examine the growth of lung cancer cells. (**C**) Colony formation assay was performed to examine the growth of lung cancer cells on soft agar. (**D**) Beta-gal staining was performed to examine the senescence of lung cancer cells; *, *P*<0.05; **, *P*<0.01.

Having shown the effects of SYT7 overexpression on the growth and senescence of lung cancer cells, we aimed to explore the functions of endogenous SYT7 by knocking down its expression in lung cancer cells (Figure 3A). Knocking down the expression of SYT7 inhibited the growth and colony formation of A549 and H23 cells (Figure 3B,C). Consistently, the number of beta-gal positively stained cells was increased upon knocking down the expression of SYT7 (Figure 3D). Taken together, these data suggested that SYT7 promoted the malignant behavior of lung cancer cells.

### SYT7 inhibited the senescence of lung cancer cells by down-regulating the expression of P16, P21, and P53

**Figure 4:**
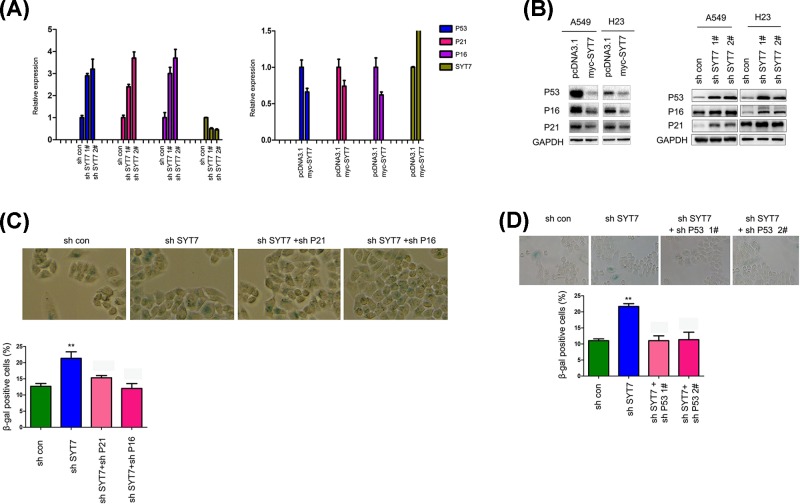
SYT7 inhibited the senescence of lung cancer cells by down-regulating P21 and P16 (**A**) Q-PCR was performed to examine the expression of P53, P21, and P16. (**B**) Western blot was performed to examine the expression of P53, P21 and P16. (**C** and **D**) Knocking down the expression of P16, P21 and P53 rescued the senescence induced by SYT7 down-regulation; **, *P*<0.01.

Having shown that SYT7 inhibited the senescence of lung cancer cells, we next explored the molecular mechanism. Knocking down the expression of SYT7 increased the mRNA levels of P16, P21 and P53, and vice versa (Figure 4A). Consistently, overexpression of SYT7 inhibited the protein levels of P16, P21 and P53, and knocking down SYT7 increased the protein levels of P16, P21 and P53 (Figure 4B). Moreover, knocking down the expression of P21, P16, and P53 abolished the senescence induced by the down-regulation of SYT7 (Figure 4C,D). Collectively, these data suggested that SYT7 inhibited the senescence of lung cancer cells by down-regulating the expression of P16, P21, and P53.

### SYT7 interacted with P53 and enhanced the interaction between P53 and MDM2

**Figure 5:**
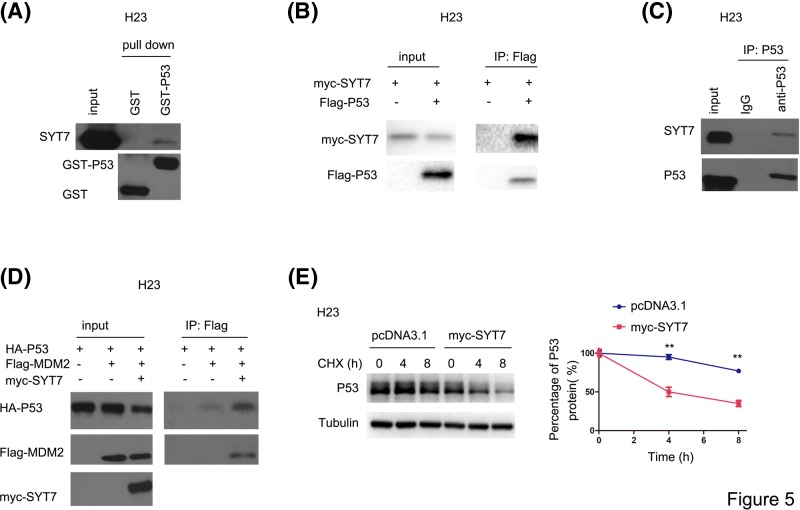
SYT7 interacted with P53 (**A**) GST pull-down was performed to examine the interaction between P53 and SYT7. (**B** and** C**) Immunoprecipitation was performed to examine the interaction between P53 and SYT7. (**D**) The effects of SYT7 expression on the interaction between P53 and MDM2 were examined. (**E**) The H23 cells were treated with CHX (10 µg/ml) for the indicated time, and the protein level of P53 was examined; **, *P*<0.01.

To study the molecular mechanism of SYT7, we examined the interaction between SYT7 and important senescence regulators. In the GST pull-down assay, the fusion protein GST-P53 interacted with SYT7 (Figure 5A). The interaction between SYT7 and P53 was also demonstrated in the immunoprecipitation assay (Figure 5B,C). Most importantly, SYT7 enhanced the interaction between P53 and its E3 ligase MDM2 (Figure 5D), suggesting that SYT7 regulated the protein stability of P53. Consistent with these observations, overexpression of SYT7 shortened the half-life of P53 (Figure 5E).

## Discussion

Although advances have been made in the diagnosis and treatment of lung cancer, the survival outcome for lung cancer is still very poor due to lung cancer metastasis and occurrence [14,15]. Therefore, it is of great significance to further investigate the molecular mechanisms of lung cancer occurrence and metastasis. Using an open-access database, we searched for genes important for lung tumorigenesis and identified an association between higher SYT7 expression and worse survival, suggesting the important function of SYT7 in lung cancer.

To assess the biological functions of SYT7 in lung cancer, MTT assay, colony formation assay and beta-gal staining of senescent cells were performed. SYT7 was shown to promote the growth and colony formation of lung cancer cells. In addition, SYT7 was found to inhibit the senescence of cancer cells. Consistent with these observations, SYT7 was overexpressed in colorectal cancer and regulates colorectal cancer cell proliferation [7], while down-regulation of SYT7 inhibited glioblastoma growth by promoting cellular apoptosis [9]. Although we did not test the effect of SYT7 on the motility of lung cancer cells, SYT7 has been recognized as the driver for the metastasization of gastric cancer.

One of the most interesting findings of the present study was that SYT7 inhibited the senescence of lung cancer cells. Cellular senescence has been recognized as a phenotype for tumor suppression [16,17]. P53, P27, and P16 are important regulators of cell senescence [18,19]. In the present study, we have shown that SYT7 interacted with P53 and enhanced the interaction between P53 and MDM2, suggesting that SYT7 promoted the degradation of P53. Consistent with this hypothesis, SYT7 decreased the protein levels of P53 and P21. In addition, it has been found that SYT7 negatively regulated the mRNA levels of P53, P21, and P16. Although we did not explore the molecular mechanism through which SYT7 inhibited the mRNA levels of P53, P21 and P16, we supposed that SYT7 inhibited the mRNA levels of P53, P21, and P16 by up-regulating TGF-β 3 based on the reports that SYT7 up-regulated the expression of TGF-β 3 and TGF-β 3 inhibited the mRNA levels of P53 [20,21].

Taken together, the present study revealed the oncogenic role of SYT7 in lung cancer and suggested that SYT7 might be a good therapeutic target for lung cancer treatment.

## Abbreviations

DAB: Diaminobenzidine
IHC: immunohistochemistry
MDM2: Mouse double minute 2
MTT: 3-(4,5-methylthiazol-2-yl)-2,5-diphenyltetrazolium bromide
NP40: Nonidet P40
RIPA: Radio Immunoprecipitation Assay
SYT7: synaptotagmin-7
TBST: tris-buffered saline with tween 20
